# Cardiovascular disease in transgender people: recent research and emerging evidence

**DOI:** 10.1093/cvr/cvab288

**Published:** 2021-09-24

**Authors:** Paul J Connelly, Christian Delles

**Affiliations:** Institute of Cardiovascular & Medical Sciences, University of Glasgow, BHF GCRC, 126 University Place, Glasgow G12 8TA, UK

**Keywords:** Hormone, Gender, Transgender, Androgen, Blood pressure

People who are transgender have gender identities that differ from the sex they were assigned at birth. Although accurate data relating to the global size of the transgender population is lacking, estimates suggest a prevalence of 0.5%, which may account for as much as ∼25 million individuals worldwide.^1^ In healthcare systems that facilitate access to gender-affirming healthcare, these individuals may commence gender-affirming hormone therapy (GHT), which aims to align their secondary sex characteristics to their gender identity. In transgender men, this comprises of testosterone administration to achieve levels in the male range, while transgender women receive oestrogens, which may be prescribed in combination with an anti-androgens (e.g. gonadotrophin-releasing hormone analogues or cyproterone acetate).

Sex steroid receptors are ubiquitous in the vasculature and play an important role in the sexual dimorphic modulation of cardiovascular risk in cisgender individuals (i.e. those whose sex aligns with their gender identity).^2^ Consequently, it is anticipated that GHT may modulate the development of cardiovascular disease in transgender individuals, yet data relating to cardiovascular outcomes in transgender people remains limited. This is reinforced by the methodological and ethical barriers of undertaking research in this population, such as the inability to assess GHT causality via randomized control trials.^3^

Hypertension remains the leading modifiable risk factor resulting in cardiovascular disease, and sex steroids are believed to regulate blood pressure (BP) and mediate sex differences in this condition.^2^ We recently published a systematic review concluding that there is insufficient data to advise the impact of GHT on BP. In this review, we identified 14 uncontrolled pre–post studies comprising of 1309 transgender individuals.^4^ Generally, these studies were heterogeneous in their interventions, lacked outcome uniformity, and were underpowered to detect significant differences in BP. Consequently, even fundamental aspects of cardiovascular health remain uncertain in transgender populations.

The methodological issues highlighted in this study are evident throughout the literature. However, recent engagement with the transgender population in cardiovascular research has broadened our understanding of putative cardiovascular effects associated with GHT and directed researchers towards providing evidence-based care for these individuals with the aim of ameliorating that risk.

With respect to BP, Banks *et al*.^5^ have recently published a study of 470 transgender individuals with multiple BP readings for up to 57 months following the initiation of GHT. This is the largest and longest observational study to date of this type and demonstrated that within 4 months of commencing GHT there were sustained increases in systolic BP (2.6 mmHg) in transgender men, and a decrease in systolic BP (4 mmHg) in transgender women. No changes in diastolic values were observed in either group. Interestingly, there appears to be interindividual variation in the response to GHT, where ∼25% of transgender women experienced ≥5 mmHg increase in systolic or diastolic BP, whereas equal proportions of transgender men experienced a decrease. These data support the routine BP monitoring of transgender individuals and provide an evidence base to do so. It also sets forth many additional research questions relating to the cardiovascular health in transgender people: What are the longer-term effects of GHT? How does age interact with GHT to modulate BP? What BP targets should we aim for in transgender men and women? And, what are the mechanisms responsible for BP variation? These will only be answered through rigorous, well-conducted research and involvement of the transgender population.

The Behavioural Risk Factor Surveillance System (BRFSS) has also offered much needed insight into the understanding of ‘hard’ cardiovascular outcomes in transgender populations.^6^ The BRFSS analysis of myocardial infarction (MI) in people who are transgender between 2014 and 2017 demonstrated that transgender men had a greater than four-fold risk of MI compared to cisgender females after adjusting for cardiovascular risk factors. This association was not observed in transgender women. There are many limitations evident in studies of this type: they are cross-sectional and therefore associational; outcomes are self-reported and subject to recall bias; and there is no assessment of GHT use. However, this study does provide significant value in compiling data directly from over 3000 transgender individuals and produces national prevalence estimates.

Many of these limitations can be addressed through population research utilizing electronic health records. Recently, Getahun *et al.*^7^ utilized the Kaiser Health system to conduct a large observational analysis of transgender health outcomes. Unlike the BRFSS analysis, this did not show any increase in MI in transgender men. This difference may be a consequence of the Kaiser study’s significantly younger cohort, the definition of transgender status being imputed by healthcare providers rather than self-reported, and lastly the Kaiser study only included transgender individuals who are insured, which is unlikely to be representative, compared to the BRFSS that is designed through random sampling to be typical of the US population. These discrepancies highlight the heterogeneous nature of this population and the need for inclusivity in study design. Interestingly, this study did demonstrate an increased risk of ischaemic stroke in transgender women, which merits further investigation.

In addition to these outcome data, research is emerging on increased adverse social determinants of cardiovascular health in this population. Factors such as mental health disorders, substance misuse, and health inequalities undoubtedly contribute to the burden of cardiovascular risk.^8^ Smoking is an established modifiable risk factor in the development of cardiovascular disease. Data from the 2015 US Transgender Survey, an online survey undertaken by the National Centre for Transgender Equality with ∼27 000 respondents over the age of 18, have demonstrated that 23.6% used cigarettes, 9.3% used e-cigarettes or vaping products, and 5.2% reported use of either within the previous month.^9^ This demonstrates a higher burden of tobacco use compared to the wider US population, of which 17.6% smoke. Almost half of the respondents experienced some form of discrimination, and importantly experiencing multi-modal discrimination (i.e. unequal treatment, verbal harassment, and physical assault) doubled the risk of smoking compared to those who had not faced such unjust treatment. Consequently, socioeconomic inequalities that infer greater cardiovascular risk may be more pervasive in the transgender population.

A recent American Heart Association Scientific Statement ‘Assessing and Addressing Cardiovascular Health in LGBTQ Adults’ highlighted the opportunities for research to better understand and ameliorate cardiovascular risk in this underserved population.^10^ As demonstrated in this article, significant progress is being made, however, clear gaps exist in our understanding of the relationship between being transgender and cardiovascular health, and particularly the role of GHT. Potential avenues for research include prospective cardiovascular longitudinal studies and the incorporation of transgender individuals into existing cardiovascular trials. There is a lack of mechanistic research, particularly addressing the consequences of sex-chromosome and -steroid interaction, or cross-reaction, in this population. Lastly, we must also consider the impact of societal and economic discrimination on these individuals and the effects this has on health outcomes. Progress can only be achieved through the development of a research strategy that is both inclusive to transgender participants and their health priorities.

## Funding

The current work was supported by the British Heart Foundation (Centre of Research Excellence Awards RE/13/5/30177 and RE/18/6/34217).

**Conflict of interest:** None declared.

## Figures and Tables

**Figure:**
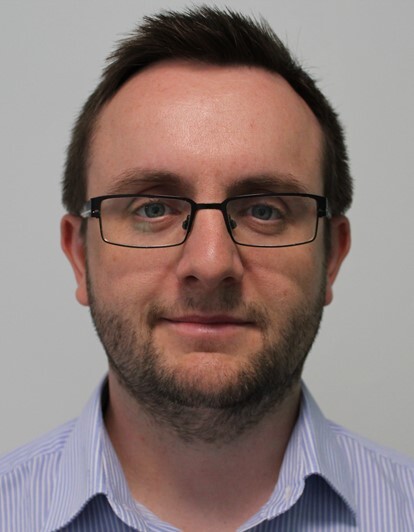


**Figure:**
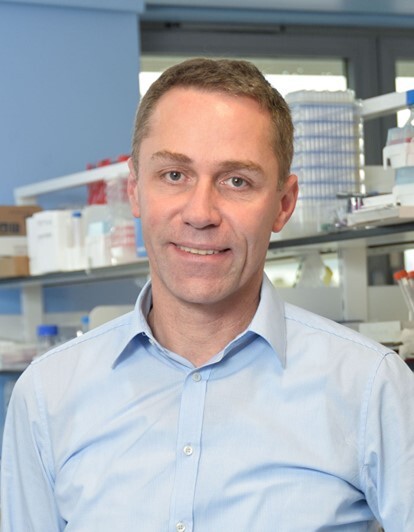


## Authors

**Biography:** Dr Paul J. Connelly is a clinical research fellow and endocrinology registrar at the BHF Centre of Research Excellence, University of Glasgow. He studied medicine and pharmacology at the University of Dundee before moving to Glasgow to complete his clinical training and undertake a PhD under the supervision of Professor Christian Delles and Professor Rhian Touyz. His research interests include the relationship between sex, gender, and cardiovascular disease, with a particular focus on transgender health, and cardiovascular endocrinology.

**Biography:** Prof Christian Delles is a Professor and Acting Director at the Institute of Cardiovascular and Medical Sciences, University of Glasgow. He studied medicine in Freiburg, Germany, and Innsbruck, Austria, and trained in internal medicine in Erlangen and Nürnberg, Germany, before moving to Glasgow in 2003. He is interested in cardiovascular risk factors and how they translate into overt cardiovascular disease. His translational research focuses on hypertension, renal diseases, and particularly diabetic nephropathy, pregnancy-associated conditions such as pre-eclampsia and gestational diabetes, and the role of sex and gender in the development of cardiovascular diseases. He is also an Associate Editor of *Cardiovascular Research*.
